# Editorial: Targeting MYCN in Pediatric Cancers

**DOI:** 10.3389/fonc.2014.00330

**Published:** 2015-05-13

**Authors:** Arturo Sala

**Affiliations:** ^1^Brunel Institute of Cancer Genetics and Pharmacogenomics, College of Health and Life Sciences, Brunel University London, London, UK

**Keywords:** neuroblastoma, paediatric cancer, MYC, MYCN, tran

MYCN is the product of a gene frequently deregulated in childhood tumors that belongs to a small but very famous family of transcription factors whose prototype member is c-MYC. The other member of the family is L-MYC, identified as a gene amplified in a subset of lung cancers. c-MYC is widely expressed in normal tissues, is the most deregulated protooncogene in human cancer, and not surprisingly, it is the subject of intensive investigations in many laboratories worldwide. Clearly, decoding its function in tumorigenesis and finding ways of inhibiting its oncogenic activity would have a very large impact in terms of human health. In contrast to the broad significance of c-MYC, the expression of MYCN is temporally and spatially restricted during embryonal development, being detected mostly in cells of the developing nervous system. This more limited function is also reflected in human pathology, with only a few types of tumors presenting alterations of MYCN. These cancers arise in the nervous system, both central and peripheral, manifesting as medulloblastomas, gliomas, and neuroblastomas. Despite the fact that MYCN-positive tumors are relatively rare, their very aggressive nature and the pediatric setting make therapeutic treatments a clinical challenge.

Increased expression of MYCN can be observed in cancers in the absence of overt aberrations of its gene structure, but about half of high-risk, metastatic neuroblastomas are characterized by amplification of the MYCN gene, leading to high mRNA and protein expression in tumor cells. Amplification of MYCN is also detected in neoplasias of the central nervous system, although at lower frequency than neuroblastoma. Importantly, it is now well established that MYCN is a direct driver of pediatric cancers: transgenic expression of MYCN in the neuroectoderm, the sympathetic tissue from which neuroblastoma originates in humans, or the cerebella that causes the development of neuroblastomas and medulloblastomas in mice (1, 2). Furthermore, other oncogenes have been shown to transform cells of the nervous system by enhancing the expression of MYCN or by stabilizing its protein product (3–5). The central role of MYCN in pediatric cancers renders it an ideal candidate for gene therapy, if specific inhibitors be developed. Unfortunately, many years of research from the private and academic sectors have made evident that it is extremely difficult to find small molecule inhibitors targeting transcription factors such as c-MYC and MYCN. An answer to this almost intractable problem could be the use of drugs that indirectly affected the transcriptional activity of MYCN. For example, MYC gene expression depends on the activity of the co-factor bromodomain and extra-terminal (BET) family member BRD4 that can be inhibited by a cell permeable compound called JQ1. Different research teams have demonstrated that tumors with deregulated MYCN are susceptible to JQ1 inhibition *in vitro* and *in vivo* (6, 7). The MYCN protein requires the activity of the PI3K kinase pathway for stability. A research paper in this topic shows that newly engineered PI3K inhibitors, PIK-75 and PW-12, are able to destroy the MYCN protein in mouse models of neuroblastoma and medulloblastoma, suggesting that they might be developed into useful drugs for these cancers (8). Another approach is to dissect the signaling pathways that lie downstream and upstream of MYCN, trying to illuminate the gene networks required for homeostasis of tumor cells with deregulated MYCN. Indeed, increased MYC expression is known to induce oncogenic stress that requires the balancing activity of a plethora of factors that can be exploited pharmacologically to induce “synthetic lethality.” In this regard, several papers in the research topic describe MYCN-activated genes [i.e., MDM2, SKP2, ornithine decarboxylase, ATP-binding cassette (ABC) transporters] that could be used as potential targets for therapy (9–12). Another interesting aspect of MYCN biology highlighted in the papers from the Perini’s and Thiele’s laboratories is that the transcription factor is not only an activator of gene expression but also a mediator of transcriptional repression (13, 14). The most recent view is that this activity might be as important as that of transcriptional activation in tumorigenesis. Several putative tumor suppressors have been identified as MYCN-repressed genes, including p75, TRKA, CASZ1, and clusterin (15–18). Notably, transcriptional repression is also achieved by MYCN via epigenetic modifications, suggesting that epigenetic drugs could be used in the clinic to successfully treat MYCN-amplified tumors. In this regard, preliminary evidence *in vitro* and *in vivo* indicates that histone deacetylase inhibitors or small molecule drugs targeting the histone methyltransferase EZH2 might be useful in the context of MYCN-amplified tumors (16, 18).

Last, but not least, MYCN could serve as a tumor-associated antigen given that its expression is only marginal in postnatal tissues. Overexpression of the MYCN protein outside the embryonal context could induce a break in the immunological tolerance and expose tumor cells to the attack of the immune system. The paper by the Pistoia’s team describes the possible role of MYCN as a tumor-associated antigen and strategies to generate cytotoxic T cells directed against pediatric tumors expressing the oncogene (19). Immunological therapy with the antibody against tumor-associated disialoganglioside GD2 has shown some success in inducing remission in children with relapsed metastatic neuroblastomas (20). Other forms of immunotherapy in clinical development are the generation of chimeric antigen receptor-engineered T cells that can be directed against tumor cells expressing the GD2 antigen such as neuroblastoma, sarcoma, and melanoma. One could speculate that MYCN is an even better antigen for immunotherapy because it is almost exclusively expressed by tumor, but not normal, cells.

Despite pediatric cancers bearing activation of MYCN are extremely malignant, their dependence on this oncoprotein for proliferation and survival makes them potentially curable diseases when more efficient ways of gene targeting will be developed.

## Conflict of Interest Statement

The author declares that the research was conducted in the absence of any commercial or financial relationships that could be construed as a potential conflict of interest.
